# Parasystole in a Mahaim Accessory Pathway

**Published:** 2014-07-15

**Authors:** Roopa Tekkatte, Ahmed Hamoud, Claire Warren, James Barry

**Affiliations:** 1 Cardiff and Vale University Health Board, United Kingdom; 2 Abertawe Bro Morganng NHS Trust, United Kingdom

**Keywords:** Inappropriate Therapies, Atrial Flutter, Subcutaneous ICD

## Abstract

Hypertrophic cardiomyopathy's (HCM) association with sudden cardiac death is well recognised. The risk of sudden cardiac death is known to increase when there is a history of unexplained syncope, abnormal blood pressure response during exercise, severe left ventricular hypertrophy or a family history of unexplained death.

Implantable Cardioverter Defibrillator (ICD) implantation has been widely used for primary and secondary prevention of sudden cardiac death (SCD) in people with HCM. Subcutaneous ICD (S-ICD) therapy has been developed to overcome some of the problems associated with the transvenous leads used in conventional ICDs.

In this article, we report the use of S-ICD in a patient with HCM and multiple risk factors for sudden cardiac death, this device had to be extracted due to recurrent inappropriate shocks caused by over sensing of atrial flutter and failure to treat a VT episode. We are not aware of any reports of inappropriate shocks caused by atrial flutter in people with a S-ICD.

## Case Details

An 18 years old male was referred to the regional cardiac centre following admission to a peripheral hospital with exertional syncope during weight lifting exercises. He had no significant past medical history and he was not taking any regular medication. There was a history of sudden cardiac death in his family.

**Figure 1:**
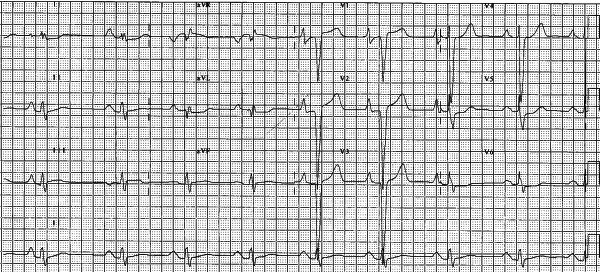
Resting 12 lead ECG demonstrating large amplitude p waves indicating atrial enlargement particularly in the precordial leads

The patient's resting electrocardiogram had shown features consistent with left ventricular hypertrophy and repolarisation abnormalities (Figure 1).

His full blood count, kidney function and inflammatory markers were all normal.

Echocardiography was performed, and the findings were consistent with a HCM phenotype with interventricular septal hypertrophy in excess of 3cms. Stress testing demonstrated a good exercise capacity but an absolute absence of systolic blood pressure increase. In view of the patient's risk factors for SCD he was counselled with regards to the benefits of ICD therapy. Due to his young age and concerns about the potential for complications during long term endovascular device therapy a subcutaneous Cameron Health ® ICD (S-ICD) was advised and implanted under general anaesthesia.

In preparation for the implant the patient underwent assessment of his surface ECG QRS morphology as per Cameron Health's recommended protocol to guard against over sensing of T waves and under sensing. The patient was discharged home on beta blockade and the device set with a VF detection zone of 240bpm.

**Figure 2:**
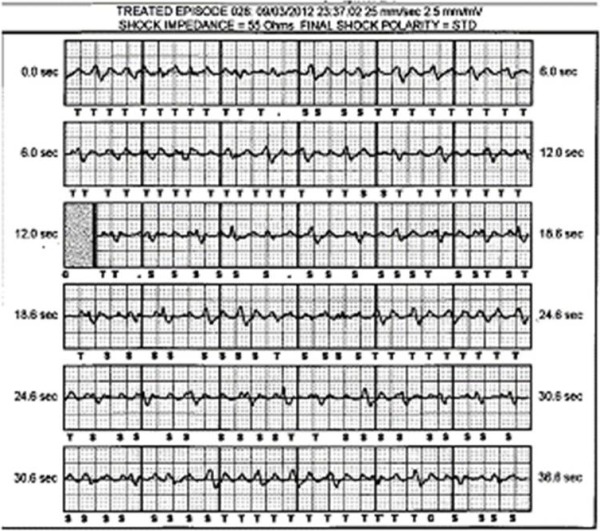
S-ICD downloads demonstrating 'over sensing' of atrial flutter

The patient was re-admitted to hospital one month following implantation, because of a conscious shock from the device. This had been associated with presyncope. The logged arrhythmia downloaded from the device demonstrated atrial flutter (Figure 2). Device interrogation demonstrated three episodes of flutter in total, only one of which received inappropriate shock therapy. The conscious shock was attributed to the over sensing of flutter waves as short RR intervals.

An attempt was made to perform a flutter ablation during this admission, this was not successful because it was not possible to produce cavo-tricuspid isthmus bidirectional block. The device was re-programmed and the setting was changed a VF zone at >250 beats per minute. The patient was loaded with oral amiodarone in addition to the pre existing bisoprolol prescription.

**Figure 3:**
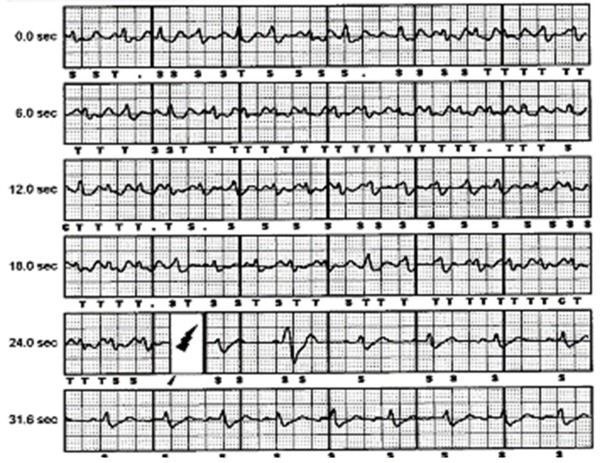
Demonstrating oversensing of P and T waves resulting in an inappropriate shock therapy

He was re-admitted to hospital three months later following an S-ICD discharge; this occurred without any preceding symptoms. Interrogation of the device demonstrated four logged episodes in the VF zone, three of which were atrial flutter associated with over sensing of flutter waves, the fourth episodes demonstrated over sensing of both P waves and T waves whist in sinus rhythm (Figure 3) The device sensing configuration was changed from the primary vector to the secondary vector in an attempt to eliminate the over sensing in sinus rhythm that was felt to be positional.

**Figure 4:**
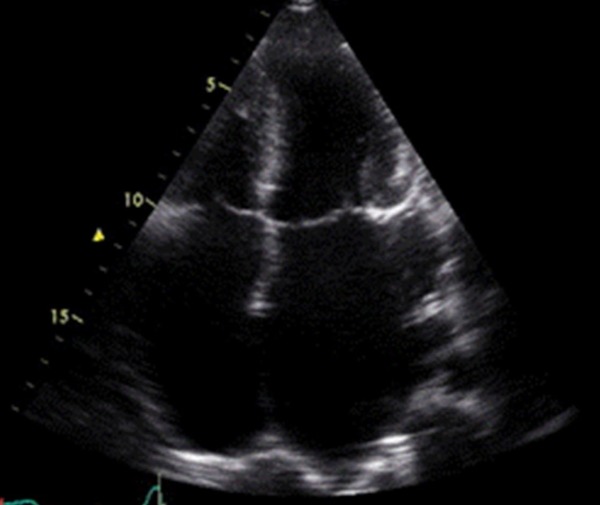
Echocardiogram in follow up demonstrating bi atrial enlargement

Echocardiography done a month before admission had shown HCM with massive bi-atrial enlargement and pulmonary hypertension (Figure 4). On review of previous echocardiograms this had been present previously.

The patient was referred to the national HCM clinic and it was advised that the device should be changed for an endovascular device. It was decided not to attempt a further flutter ablation.

**Figure 5:**
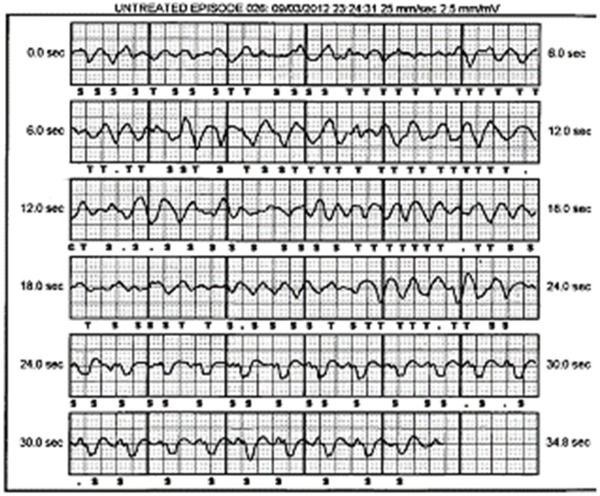
Polymorphic VT on device download

The patient was admitted for a fourth time following syncope and a subsequent conscious ICD shock. Device interrogation for logged events showed episodes of polymorphic VT (Figure 5) and atrial flutter. The polymorphic VT had been undetected and not treated. The shock had been delivered once again in response to over sensing of a flutter wave.

During this admission S-ICD was explanted and implanted with conventional ICD.

## Discussion

Implantable cardioverter defibrillator (ICD) has been considered useful and effective in prevention of fatal ventricular arrhythmias.

Conventional transvenous ICDs have been observed to have short and long term complications. These include cardiac perforation, haemothorax and pneumothorax during implantation, lead displacement and failure, system erosion and lead prosthetic endocarditis. The S-ICD has been developed to overcome these disadvantages of conventional ICD therapy where anti tachy pacing, brady pacing or resynchronisation is not required.

Previous clinical trials on S-ICD therapy have shown that it is equal or better in comparison to transvenous ICD with regards to efficacy and safety [1]. We are not aware of any previous reports regarding inappropriate shocks secondary to atrial flutter by an S-ICD.

The START study [2] compared the ability of S-ICD with single chamber and dual chamber transvenous ICD to sense arrhythmias induced during programmed electrophysiological testing in 64 patients. This demonstrated that the S-ICD specificity in discriminating supraventricular arrhythmias was superior to transvenous systems (98.0% vs. 68.0% p<0.001).

Small follow up studies have shown inappropriate therapy rates of between 3 -13 % [4,5].

The EFFORTLESS S-ICD registry continues to collect data on real world experience of S-ICD therapy and has reported a 7 % incidence of inappropriate therapies in a cohort of 369 patients during a five year follow up period. Over sensing (62%) was found to be the most common cause of inappropriate shocks.

This compares favourably to the MADIT II trial which reported 11.5% of 719 transvenous ICD patients had one or more inappropriate shocks. Most commonly this was secondary to atrial fibrillation (44%) or supraventricular tachycardia (36%). One fifth was attributed to T wave over sensing. The EFFORTLESS S-ICD registry is not obligatory. The Praetorian [3] study will further clarify the role of S-ICD therapy as a potential alternative to transvenous ICDs.

We have presented a case of inappropriate therapy for over sensing in both sinus rhythm and atrial flutter. The device did not treat an episode of polymorphic VT; however it is important to consider that this rhythm was self terminating and a traditional ICD programmed as per the MADIT-RIT [8] parameters similarly would avoided delivering a shock.

## Conclusion

The wish to avoid endovascular device therapy in patients with little need for pacing and especially in the young remains compelling and we believe S-ICD remains a viable option in such patients. In this case our patient had a particularly challenging cardiac anatomy with huge atrial enlargement that was evident on his resting 12 lead ECG. In such cases with electrocardiographic evidence of atrial enlargement implanters should consider the potential for over sensing atrial arrhythmias in addition to the standard 'morphology assessment'.
